# The clinical characteristics and treatment of ovarian malignant mesoderm mixed tumor: a systematic review

**DOI:** 10.1186/s13048-022-01037-6

**Published:** 2022-09-16

**Authors:** Xin Wang, Shiyuan Wang, Shujuan Yao, Wei Shi, Ke Ma

**Affiliations:** 1 College of Traditional Chinese Medicine, Shandong University of Traditional Chinese Medicine, No. 4655, University Road, Changqing District, Jinan, Shandong 250355 PR China; 2 Department of Gynecology, Affiliated Hospital of Shandong University of Traditional Chinese Medicine, No 16369, Jingshi Road, Jinan, 250014 PR China

**Keywords:** Ovarian malignant mesoderm mixed tumor, Clinical, Pathology, Treatment, Prognosis

## Abstract

**Background:**

Ovarian malignant mesoderm mixed tumor (OMMMT) is a rare clinical entity. To provide reference for the treatment and prognosis of OMMMT, we analyzed the clinical features, pathology and molecular biology characteristic of published cases.

**Methods:**

The English and Chinese reported cases of OMMMT were selected from PubMed, Clinical Trials.gov and CNKI database from 2000 to December 15th, 2021 following the PRISMA guidelines.

**Results:**

A total of 63 literatures including 199 OMMMT cases were included. The average age of patients at diagnosis was 56.46 years, the highest incidence age was 60-65 years, and 82% of them were menopausal women. Most patients were diagnosed in FIGO III stage (59.64%). The most common symptom of OMMMT was abdominal pain (60.5%). 61.6% of patients were accompanied by ascites, while ascites was not associated with metastatic tumor and local recurrence. The CA125 of 88.68% patients increased. The most common reported carcinomatous component and sarcomatous component were serous adenocarcinoma (44.96%) and chondrosarcoma (24.81%), respectively. Initial treatment included surgery (94.97%) and taxanes-based (55.10%) or platinum-based (85.71%) chemotherapy regimens. The median survival time of patients was 20 months. Heterologous sarcoma component did not shorten life expectancy. The optimal ovarian tumor cell debulking surgery (OOTCDS), radiotherapy and chemotherapy could significantly prolong the median survival time of patients. Furthermore, platinum drugs could significantly prolong the survival time after comparing various chemotherapy schemes. Besides, the combination of platinum and taxanes was therapeutically superior to the combination of platinum and biological alkylating agents.

**Conclusion:**

The OOTCDS and platinum-based chemotherapy regimen can improve the prognosis of OMMMT. Targeted therapy might become a new research direction in the future. Since the elderly patients are the majority, the toxicity of new drugs on the elderly patients is more noteworthy.

**Supplementary Information:**

The online version contains supplementary material available at 10.1186/s13048-022-01037-6.

## Introduction

Ovarian malignant mesoderm mixed tumor (OMMMT) is a uncommon gynecological tumor, which incidence rate only accounts for about 1-3% of ovarian malignant tumors [1]. Unlike other ovarian cancers, its histological components include both cancerous components and sarcoma components. The common types of cancerous components are serous adenocarcinoma, endometrial carcinoma and clear cell carcinoma [2, 3]. The sarcoma components can be divided into ovarian homologous tissues and other non-ovarian heterologous tissues, such as endometrial stromal sarcoma, fibrosarcoma and chondrosarcoma [4].

The mean age of OMMMT at diagnosis patients is high, ranging from 60 to 70 years old. Most of the patients are postmenopausal and have fewer deliveries [5]. OMMMT has no obvious clinical signs or symptoms, which results in approximately 75% of patients being in International Federation of Gynecology and Obstetric (FIGO) stage III-IV at diagnosis [6, 7].OMMMT is more aggressive than other ovarian malignancies and has a more rapid progression. In most patients, the tumor has invaded beyond the ovary at diagnosis which results in adverse prognosis with a median overall survival of only 7 to 27 months [8]. However, the understanding of clinical features, pathological features, treatment methods and prognosis of OMMMT is limited at present because of the low morbidity rate of OMMMT. Thus, there are no specific treatment guidelines for this disease, and the best treatment option remains unclear [9]. According to the 2020 edition of the NCCN guidelines, OMMMT can be treated with carboplatin/ifosfamide, cisplatin/ifosfamide and paclitaxel/ifosfamide (Class 2B) after operation [10], but the rate of clinical benefit for patients is not high.

This research summarizes the reported cases of OMMMT from 2000 to 2021 to explore the clinical, pathology, treatment, and prognosis of OMMMT with the goal of analyzing the factors that affect the disease progression and survival time, evaluating the influence of treatment methods on prognosis, and proposing an effective therapeutic regimen of this disease in the clinic.

## Materials and methods

### Literature retrieval and data collation

Two investigators systematically searched the studies published in PubMed, Clinical Trials.gov, and China Knowledge Resource Institute (CNKI) database from 2000 to December 15th, 2021 with language restriction to English and Chinese. The search terms were ovarian malignant mixed mullerian tumor & case, ovarian malignant mixed mullerian tumor & cases, ovarian malignant mesoderm mixed tumor & case, ovarian malignant mesoderm mixed tumor & cases, ovarian carcinosarcomas & case, and ovarian carcinosarcomas & cases.

**Fig. 1:**
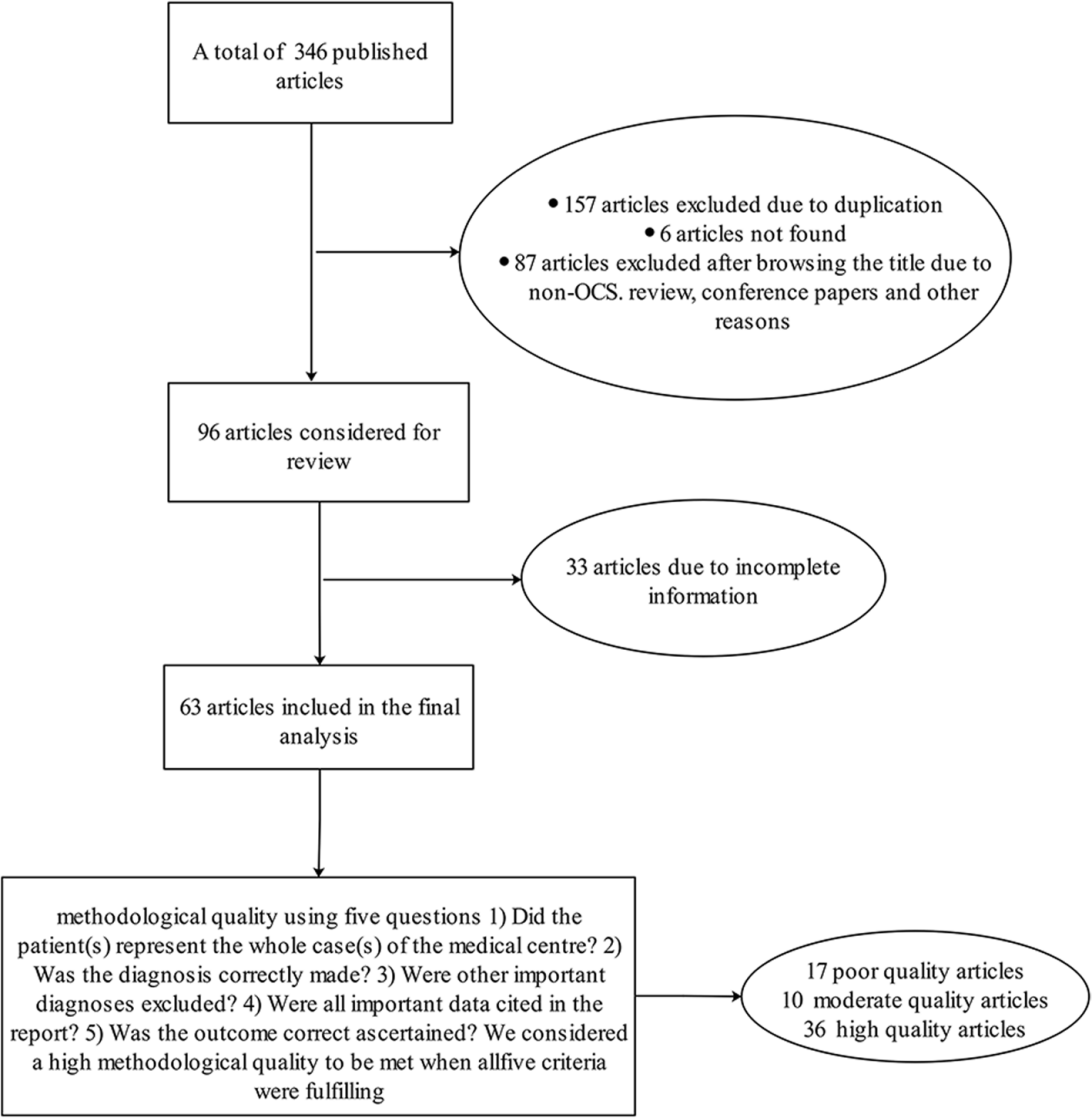
Screening process of included articles in the research

Inclusion and exclusion criteria: The article was a single-sample and multi-sample case report, which met the following conditions: ① A clear diagnosis process is presented in the article, ② Provide an individual case with medical record information. ③ Multi-case reports would be included in this study if they contained the clinical data of every individual case. Otherwise, if multi-case reports only displayed the overall characteristics of all patients, they would be defined as incomplete information and be excluded. ④ Cases from the same institution were examined and combined with their clinical information to determine if they were duplicate reports and if so, one of them would be excluded. Two investigators searched and screened articles, and when there was a dispute, the third investigator decided whether to include it in the research. The flow chart (Fig. 1) shows the identification of OMMMT and the reasons for exclusions.

The following variables were collected and recorded: (1) country; (2) age; (3) menopause or not; (4) reproductive history; (5) clinical symptoms and signs; (6) tumor site and size; (7) whether the tumor was metastatic at diagnosis; (8) surgical procedure; (9) chemotherapy or radiotherapy; (10) follow-up time, local recurrence and distant metastasis time, overall survival; (11) pathological type; (12) serum tumor marker values; (13) clinical stage; (14) other special records.

### Methodological quality

The methodological quality of the each included study was scored by five key questions (Fig. 1). This method which was proposed by Abhiram K et al. on the basis of modified the Newcastle Ottawa Scale [11].

### Statistical analyses

Data were analyzed using the Statistical Package for Social Sciences (SPSS) software (version 26.0; Ink). One-sample K-S tests were used to determine whether the data conformed to a normal distribution. The prognosis of OMMMT was analyzed retrospectively in conjunction with follow-up data. The survival analysis was performed using the Kaplan-Meier (KM) method (Log rank) for the description and estimation of survival rates. Correlation analysis was used to analyze the relationship between various factors and survival time. N-year survival rates were analyzed by the life table method. Chi-square tests were used to analyze between-group differences in categorical data. All tests were two-sided, and *P* value < 0.05 was considered statistically significant.

## Results

### Epidemiological features

**Fig. 2:**
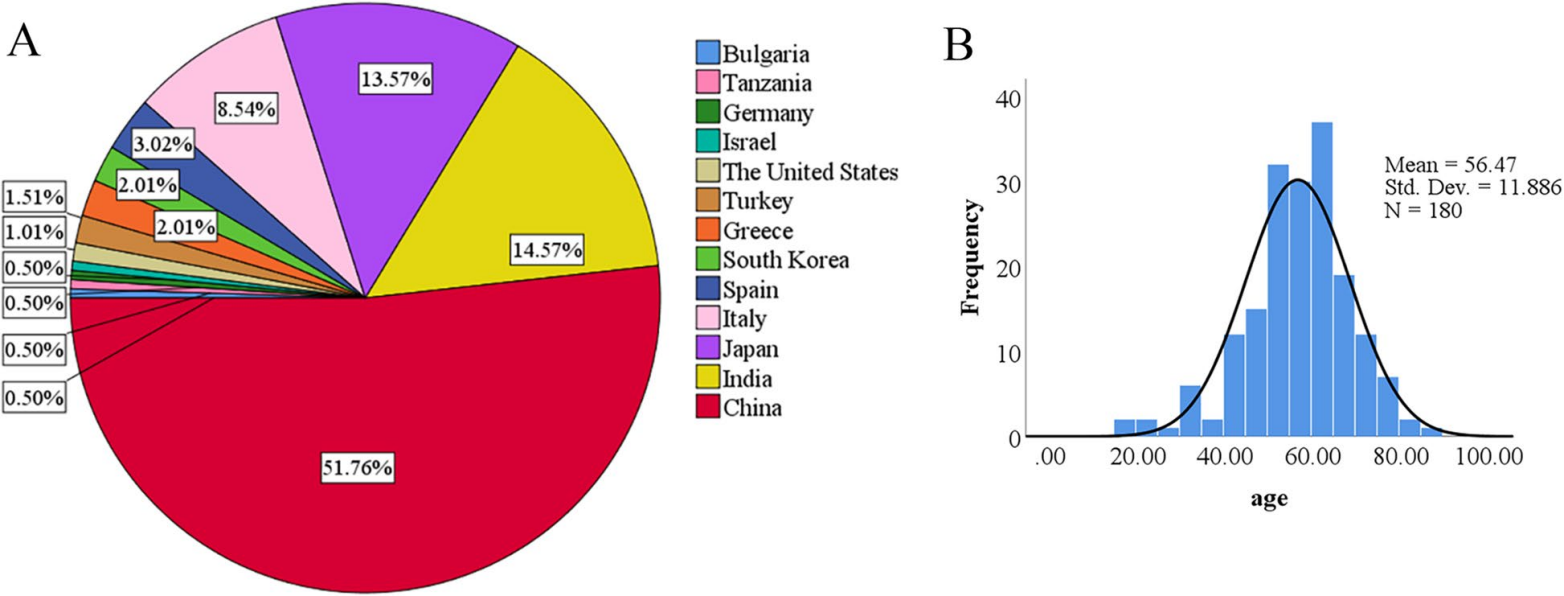
**A** Proportion of countries; **B **The age distribution of patients

Between 2000 and 2021, 63 articles met the inclusion criteria (Fig. 1), reporting 199 cases. Upon assessment of methodological quality, we deemed 17 articles to be poor quality, 10 to be moderate and 36 to be high. Patients were from 13 countries, including Japan (13.57%), India (14.57%), and China (51.76%) (Fig. 2A). Among the 180 patients, the mean age was 56.46 years at diagnosis, with a median age of 57 years, ranging from 17 to 86 years. Notably, a normality test for age revealed a right skewed distribution of patient age, indicating that the majority of the patient had a higher age and the highest prevalence age group was 60-65 years (Fig. 2B). Among the 173 patients, 142 cases (82%) were menopausal, of which 27 patients recorded the time of menopause with a mean of 8 years (Table 1). Besides, 20 patients recorded the history of pregnancy and childbirth with a mean of 2.6 pregnancies and 2.2 deliveries (Table 1). One hundred sixty-six patients described clinical stage (FIGO stage) in detail, of which 23 (13.86%) were FIGO I stage, 26 (15.66%) were FIGO II stage, 99 (59.64%) were FIGO III stage, and 18 (10.84%) In FIGO IV stage. Most patients were at a clinically advanced stage at the time of diagnosis. In addition, a correlation between OMMMT and tamoxifen administration was reported in 3 patients.

**Table 1:** Summary of the clinical and prognosis features of OMMMT patients

Subjects	No. of patients	Mean ± SD or Percentage
Age (year)	180	56.46 ± 11.89
Menopausal	142/173	82.08%
Menopausal Age (year)	27	8.06 ± 5.27
Pregnancy (time)	20	2.55 ± 1.73
Delivery (time)	20	2.20 ± 1.64
Ascites	61/99	61.61%
Tumor size (longest diameter/cm)	71	12.02 ± 6.66
Lymph node metastasis	26/82	31.7%
Pelvic and abdominal metastasis	56/76	73.68%
CA125 value	64	547.84 ± 549.15
Follow-up time (month)	166	21.40 ± 1.92
Recurrence time (month)	44	10.52 ± 1.52
Overall survival (month)	86	16.64 ± 1.74

### Clinical symptoms and signs

Clinical symptoms were recorded in 114 patients and we analyzed the frequency of various clinical symptoms. The three most frequent clinical symptoms were: abdominal mass (21.9%) abdominal distention (43.9%), abdominal pain (60.5%). Reproductive symptoms could also be manifested as vaginal bleeding (9.6%), menorrhagia (0.9%) and vaginal drainage (0.9%). A few patients might also have gastrointestinal symptoms such as anorexia (3.5%) and urinary symptoms such as frequent micturition (3.5%) due to tumor compression. In addition, systemic symptoms such as fatigue (1.8%) and loss of weight (4.4%) were seen (Table 2). Ninety-nine patients’ signs were recorded, of which, 61 (61.61%) were accompanied by ascites (Table 1). We analyzed the correlation between ascites and metastases, tumor recurrence and distant metastases respectively, and the results showed *P* > 0.05. Thus, ascites is not a significant risk factor.

**Table 2:** Frequency of various symptoms of patients

Symptom	Frequency	Percentage
Abdominal pain	69	60.5%
Abdominal distention	50	43.9%
Abdominal mass	25	21.9%
Vaginal bleeding	11	9.6%
Weight loss	5	4.4%
Anorexia	4	3.5%
Frequent micturition	4	3.5%
No obvious symptoms	4	3.5%
Abdominal circumference enlargement	3	2.6%
Dysuria	3	2.6%
Fever	2	1.8%
Constipation	2	1.8%
Fatigue	2	1.8%
Edema of lower extremity	2	1.8%
Nausea	1	0.9%
Inguinal hernia	1	0.9%
Diarrhea	1	0.9%
Straining feeling in anus	1	0.9%
Cough	1	0.9%
Dyspnea	1	0.9%
Menorrhagia	1	0.9%
Urgency of micturition	1	0.9%
Less urine	1	0.9%
Giddy	1	0.9%
Chest distress	1	0.9%
Hydrothorax	1	0.9%
Vaginal drainage	1	0.9%

### Tumor characteristics

In 71 cases, the size of the tumor was described. The average of longest diameter of the tumor was 12.02 cm (Table 1). One hundred sixteen cases recorded the primary site of the tumor: 33 (28.4%) on the right side, 44 (37.9%) on the left side, and 35 (30.2%) bilaterally. Eighty-two patients had lymph node biopsy or debulking, and 26 (31.7%) had lymph node metastasis. The presence or absence of pelvic and abdominal metastasis was summarized in 76 patients. Metastatic tumor in the pelvic or abdominal cavity could be found in 56 (73.7%) cases, and the common metastatic sites were the greater omentum (46.15%), peritoneum (40.38%), and fallopian tubes (28.85%).

### Pathology type

The carcinomatous and sarcomatous components of 129 patients were described in detail. The frequencies of different pathological types were counted in order to obtain the high prevalence of carcinomatous and sarcomatous components. The top three carcinomatous component were: serous carcinoma adenocarcinoma (58/129), endometrioid adenocarcinoma (54/129), and clear cell carcinoma (18/129) (Table 3), and the top three sarcomatous components were: chondrosarcoma (32/129), rhabdomyosarcoma (18/129), and undifferentiated sarcoma (13/129) (Table 4). Squamous cell carcinoma, neuronal differentiation, spindle cell carcinoma, angiosarcoma, and sarcoma with neuroendocrine differentiation were much rarer (Tables 3 and 4).

**Table 3:** Frequency of different carcinomatous component pathological types

Pathology type	number	Percentage
Serous carcinoma adenocarcinoma	58	44.96%
Endometrioid adenocarcinoma	54	41.86%
Clear cell carcinoma	18	13.95%
Mucinous adenocarcinoma	4	3.10%
Squamous cell carcinoma	3	2.33%
Fusiform cell carcinoma	2	1.55%
Neuron differentiation	2	1.55%
Neuroendocrine differentiation	1	0.78%

**Table 4:** Frequency of different sarcomatous component pathological types

Pathology type	number	Percentage
Chondrosarcoma	32	24.81%
Rhabdomyosarcoma	18	13.95%
Undifferentiated sarcoma	13	10.08%
Fibrosarcoma	13	10.08%
Osteosarcoma	12	9.30%
Endometrioid stromal sarcoma	11	8.53%
Leiomyosarcoma	10	7.75%
Spindle cell sarcoma	5	3.88%
Liposarcoma	4	3.10%
Hemangiosarcoma	2	1.55%

### Molecular biology

CA125 is one of the important female tumor markers. In the included study, 64 individuals provided specific values of CA125. And the mean value of CA125 was 547.84 U/ml. One hundred six patients recorded whether CA125 was within the normal range, and 88.68% of patients had abnormal CA125 levels. Besides, 40.48% of the 42 cases who provided immunohistochemical results were positive for P53 expression.

### Treatment

**Fig. 3:**
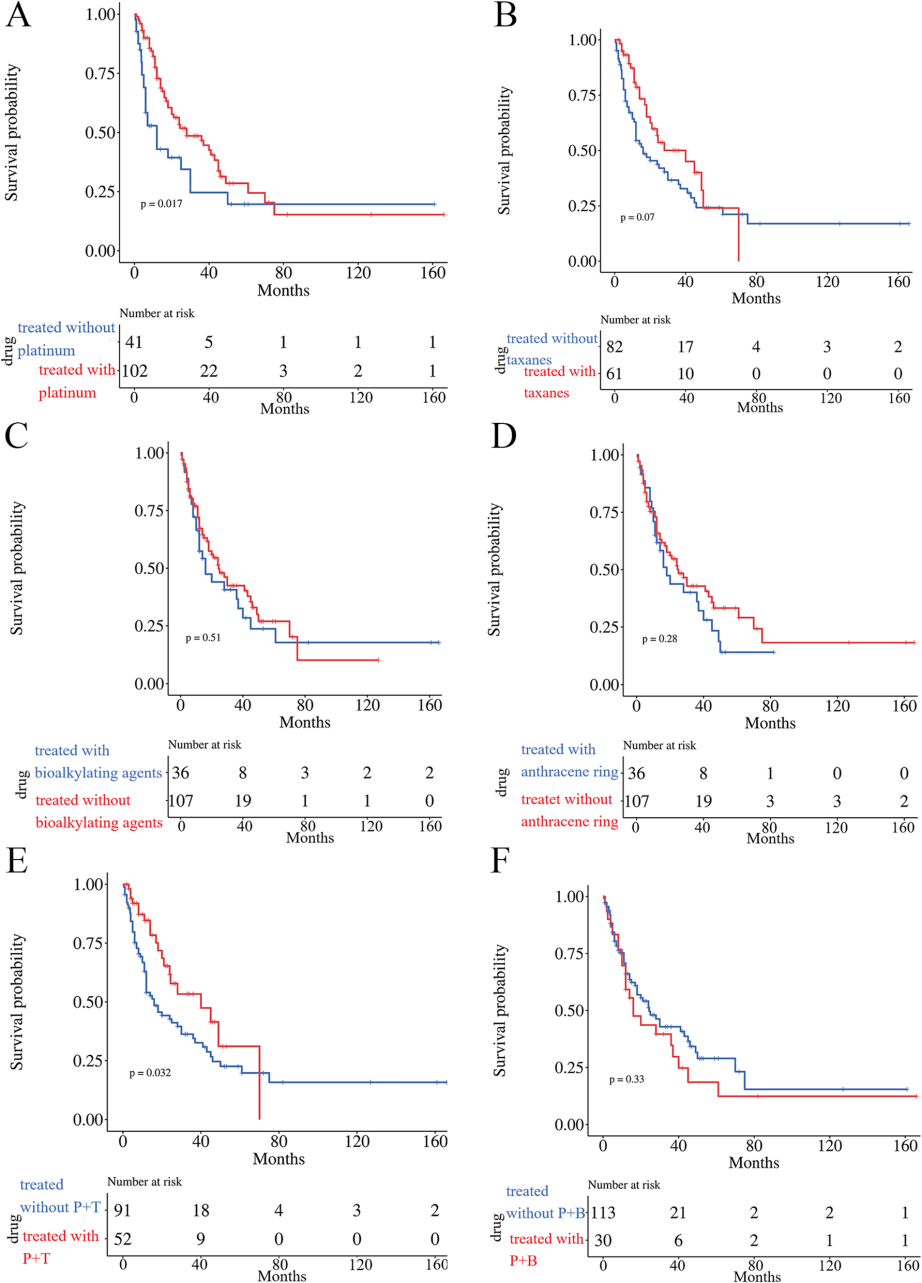
The KM Analysis of Chemotherapy Efficacy. **A** Platinum; **B** Taxanes; **C** Bioalkylating Agengts; **D** Anthracene Ring; **E** Platinum+ Taxanes; **F** Platinum+ Bioalkylating Agengts

Except for 3 patients who were unable to determine whether to undergo surgery and 7 patients who did not undergo surgery, all patients underwent surgery. The optimal ovarian tumor cell debulking surgery (OOTCDS) included total hysterectomy, bilateral oophorectomy and salpingectomy, appendectomy, omentectomy, pelvic and abdominal para-aortic lymph node dissection and other tumor volume reduction. Seventy-two of these patients underwent the optimal surgical procedure, and the remaining 118 patients underwent the suboptimal surgical procedures of total hysterectomy + bilateral oophorectomy and salpingectomy, total hysterectomy + bilateral oophorectomy and salpingectomy + omentectomy, or other surgical procedures. Twenty of the one hundred ninety-nine patients could not be determined whether postoperative radiotherapy or chemotherapy was administered. Of the remaining 179 patients, 28 did not choose radiotherapy or chemotherapy, 4 patients received radiotherapy, and the rest cases received chemotherapy. We counted the high-frequency drugs used by patients who received chemotherapy, and the results showed that bioalkylating agengts drugs (45/147), anthracene ring drugs (47/147), taxanes drugs (81/147) and platinum drugs (126/147) were commonly used drugs, such as ifosfamide, epirubicin, paclitaxel, cisplatin and carboplatin (Table 5). We compared the effects of above high frequency drugs on the survival time. The results indicated that platinum-based chemotherapy was the most effective chemotherapy scheme for the treatment of OMMMT (*P* < 0.05) (Fig. 3A). In addition, the combination of platinum and taxanes is better than the combination of platinum and biological alkylating agents. (Fig. 3E, F).

**Table 5:** Frequency of different chemotherapy drugs

Drug type	Number	Percentage
Platinum	126	85.71%
Taxanes	81	55.10%
Anthracene Ring	47	31.97%
Bioalkylating Agengts	45	30.61%
Unknown	15	10.20%
Dactinomycin-D	6	4.08%
Anti-pyrimidine	6	4.08%
Vincristine	6	4.08%
Topoisomerase inhibitor	4	2.72%
Bleomycin	2	1.36%
Pingyangmycin	2	1.36%
Targeted drug	2	1.36%
Peplomycin	1	0.68%
Platinum+ Taxanes	77	61.11%
Platinum+ Bioalkylating Agengts	38	25.85%
Bioalkylating Agengts+ Anthracene Ring	31	21.09%

### Prognosis

In order to concluded the prognosis of OMMMT, its follow-up of 199 cases was summarized. Of the 166 patients who underwent follow-up, the shortest follow-up time was 0.5 months, the longest follow-up time was 166 months, the mean follow-up time was 21.40, and the median follow-up time was 12 months. A total of 149 patients clearly reported the follow-up results at the last follow-up. Eighty-six patients were died and the mortality rate was 57.70%, with a median overall survival of 11.50 months. Forty-four cases (29.5%) reported local recurrence or distant metastasis during the follow-up period, with a mean local recurrence or distant metastasis time of 10.5 months.

**Fig. 4:**
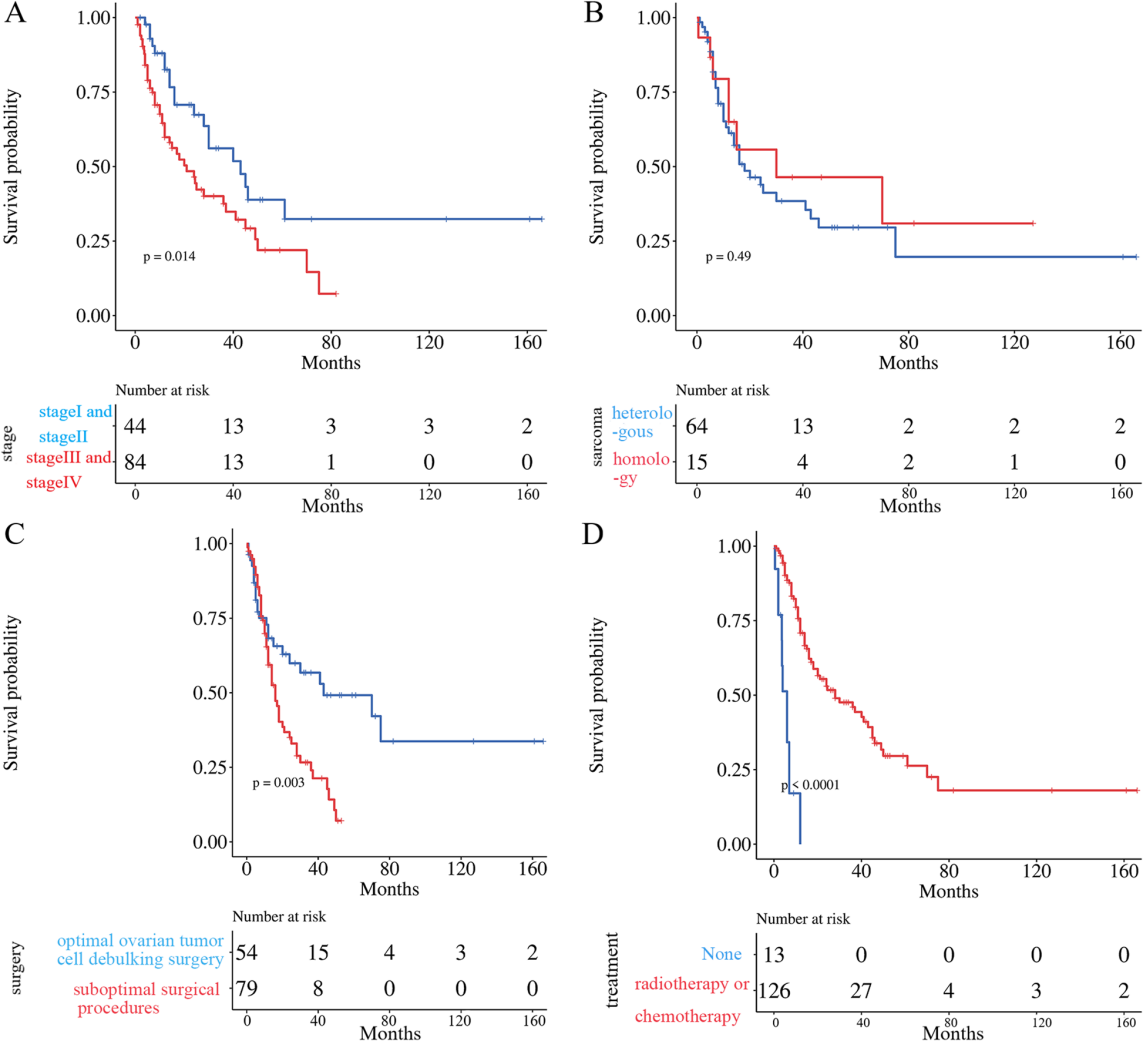
The KM Analysis of influencing factors. **A** Stage; **B** Sarcoma component; **C** Surgical modality; **D** Radiotherapy or chemotherapy

**Fig. 5:**
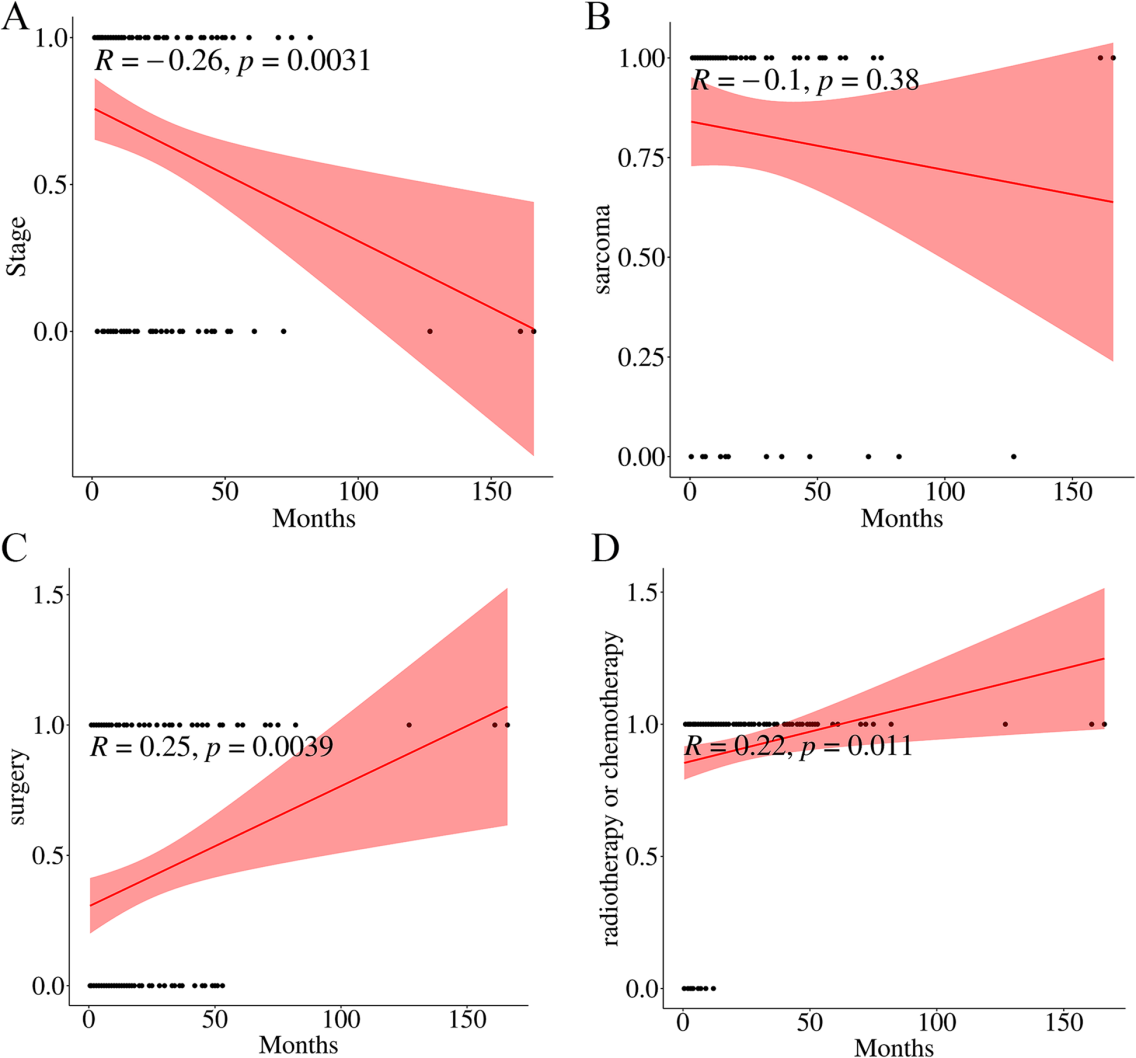
The Correlation Analysis of influencing factors. **A** Stage; **B** Sarcoma component; **C** Surgical modality; **D** Radiotherapy or chemotherapy

In addition, we analyzed the effects of clinical stage, surgical modality, radiotherapy, chemotherapy, and sarcoma component on the survival time of patients. KM and correlation analysis showed that clinical stage was related to the survival time of patients (*P* < 0.05) (Figs. 4A and 5A). Apart from this, whether the sarcoma component was homologous or heterologous tissue source had no significant effect on survival time (*P* > 0.05) (Figs. 4B and 5B).

Besides, the treatment modality had a significant impact on survival time. The results showed that OOTCDS, postoperative radiotherapy and chemotherapy could affect patient survival (*p* < 0.05) and was significantly beneficial to the patient survival time (Figs. 4C and 5C). The mean survival time was 30 months when the OOTCDS was taken. While when the suboptimal surgical procedure was executed, the mean survival time was only 16.62 months. The median survival time was also extended from 4 to 30 months in patients who adopted postoperative radiotherapy or chemotherapy compared to those who did not (Figs. 4D and 5D).

## Discussion

OMMMT is a rare female malignancy that usually occurs in postmenopausal women aged 60-70 years and 70-80% of them were being in advanced stage at the time of presentation [12]. Its most common clinical presentation is abdominal distention, abdominal pain and abdominal mass, with ascites in 67-100% of patients [13, 14], and 1/3 of cases involving bilateral ovaries [4]. These phenomena roughly consistent with our result. Abdominal symptoms cause OMMMT to be misdiagnose for gastrointestinal disease and treated. Pan et al. demonstrated that ascites did not affect the survival time of OMMMT [15]. Our study also proved that there was no correlation between ascites and metastases, tumor recurrence, distant metastases and median survival time. Several studies suggested that ovarian tumors might have a predisposition in terms of location distribution. For example, Cui et al. concluded that the incidence of malignant ovarian goiter was higher in the left ovary [16]. Another research declared that the incidence of right-sided ovarian germ cell cancer was significantly higher regardless of age [17]. Our results suggest a higher prevalence of OMMMT in the left ovary. Recent studies have proposed that tamoxifen increases the risk of developing uterine sarcoma [18, 19]. However, only three published patients in our study found that tamoxifen may contribute to the development of OMMMT [20, 21]. Consequently, there is insufficient evidence to confirm a correlation between tamoxifen and OMMMT. A number of factors were considered to be prognostic factors for OMMMT, including CA125 level [13], heterologous sarcomatous component [22], the small vessel content of the tumor tissue [23], serous epithelial component [24], the expression of vascular endothelial growth factor (VEGF) [25], the mutation of p53 [23], older age, advanced stage, and suboptimal surgical resection [26, 27]. However, there is no consensus about these factors [26]. Therefore, a systematic review is necessary to conduct to provide quantitative evidence for a relationship between these factors and the prognosis of OMMMT.

The preoperative diagnosis of OMMMT is difficult because it does not have specific tumor markers and imaging features. Some diagnostic work-up methods including pelvic ultrasound, abdominal-pelvic computed tomography (CT), chest X-ray and positron emission tomography (PET) scans are still necessary for the diagnosis of ovarian cancer [28]. Although the images features are not sufficient for diagnosis, the detailed clinical and imaging features may be helpful to improve the familiarity of this rare tumor [29]. A multicenter retrospective study suggested that the most common ultrasound image of OMMMT was a large solid tumor with irregular margins and inhomogeneous echogenicity in solid tissue and cystic areas [30]. The second most common pattern is a large, multilocular solid mass with heterogeneous echogenicity in solid tissue. However, the characteristic ultrasound signs remain undetected [30]. CT examination of patients with OMMMT found that most tumors showed unilateral large cystic solid masses with moderate or high enhancement, and the apparent diffusion coefficient of solid components was relatively high [29]. The PET/CT scan was useful for the detection of metastatic lesions, and a case of OMMMT patient who benefited from early detection of breast lymph node metastasis with PET was reported [31]. Researches suggested that CA125 was closely correlated with the prognosis of OMMMT patients [18], and study proposed that preoperative CA-125 levels exceeding 75 U/ml predicted a poor prognosis [4]. Our study showed that over 88.68% of patients had abnormal CA125 levels and the mean value was as high as 547.84 U/ml. Except CA125, some serum indicators such as β-hCG, Alpha-fetoprotein (AFP), Anti-Müllerian hormone (AMH), and LDH may also be associated with the prognosis of ovarian cancer [28]. Studies suggested that β-HCG could promote EMT and metastasis of ovarian cancer cells as well as promote tumor growth and angiogenesis, which was related to the poor prognosis of patients [32, 33]. Ovarian cancer that produces AFP is uncommon but usually has a much worse prognosis [34]. Serum LDH was regarded as a reliable marker to distinguish ovarian cancer from benign ovarian tumor, and high level of serum LDH was associated with short progression-free survival time [35]. AMH is a marker of ovarian reserve in women following anti-cancer therapy and can be used to assess the prognosis in fertility-sparing patients [28, 36]. Although the relationship between the above serum indicators and OMMMT is still unclear, its impact on prognosis can be discussed in future.

There are several different theories about the genetic origin of OMMMT, among which the most widely accepted one is the monoclonal origin theory (cancer and sarcoma components originated from a common pluripotent stem cell precursor) [37]. It had also been suggested that carcinosarcoma seemed to originate from endometriosis [38]. Many studies confirmed that endometriosis increased the risk of ovarian cancer [39]. In particular, endometrioid carcinoma and clear cell carcinoma of the ovary often occurred in the setting of combined endometriosis [40]. One article reported a case of OMMMT with a history of endometriosis and adenomyosis [41]. Therefore, whether the origin of OMMMT is related to endometriosis deserves further study. A case of OMMMT with BRAF V600E and TERT promoter mutation was reported [42]. Carnevali et al. found one OMMMT case had a family history of hereditary breast cancer-ovarian cancer syndrome and missing of BRCA1 allele, and the other case was a Lynch syndrome patient alive after 51 months with MSH6 mutation [43]. Notably, Lynch syndrome patient are seemed to have a better prognosis than other types. However, there is not enough evidence to confirm the relationship between OMMMT and hereditary cancer syndrome. Studies suggested that TP53 was positive in nearly half of OMMMT patients [15], and our study also showed that 40.48% of patients were positive in TP53 immunohistochemistry. In addition, a meta-analysis of the mutation type of TP53 gene in uterine carcinosarcoma revealed that low-copy-number/TP53-wild-type was dominant, while subgroup with high mutation burden (Pole/Ultra Mutated and microstatelitenstable/Hypermutated) was less common [44]. Tang et al. demonstrated that the mutation of Kirsten Rat Sarcoma Viral Oncogene Homolog and TP53 deletion in mouse ovarian epithelial cells could induce OMMMT. Its epithelial component was mainly endometrioid carcinoma, and the tumor was easy to metastasize, and the risk of death was obviously increased [45]. Study suggested that excessive mutations in the H2A and H2B gene encoding the histone in OMMMT increased the expression of EMT markers as well as tumor migration and invasiveness, which might play a role in promoting epithelial component transformation into sarcomatous component [46]. A decrease in the expression of E-cadherin and β-catenin reduces cell adhesion and they were associated with metastasis and invasion of carcinoma cells and a poor survival rate in patients with various types of carcinomas [47, 48]. The negative expression of both E- cadherin and β -catenin in the sarcomatous component in OMMMT cells was confirmed. Besides, the loss of cell adhesion in the sarcomatous component might resulted in the stronger aggressive and metastatic properties of OMMMT cells [49]. Human epidermal growth factor receptor 2(HER2) regulates the growth, survival and differentiation of tumor cells through the activation of kinase-mediated downstream signaling pathway [50, 51]. Cancer with HER2 overexpression has a poor prognosis which includes higher mortality from early disease, a shorter duration of recurrence, and a higher incidence of metastasis [52, 53]. Previous studies demonstrated that HER2 was highly expressed in some uterine carcinosarcoma [54, 55]. For example, HER2 overexpression was found in 7 of 24 uterine carcinosarcoma cases (29.2%) studied by immunohistochemistry [56]. In a recent study, HER2 protein overexpression and gene amplification were detected in 25% of primary OMMMT cell lines. The combination of VEGF with endothelial cell-specific receptors promotes cell division, proliferation, and tumor angiogenesis. There were evidences indicated that overexpressed VEGF in ovarian and uterine carcinosarcoma affected tumor progression and poor prognosis [25]. Another study with 9 OMMMT cases found that overactivity for VEGF and memory T cells was observed in four and two OMMMT tumor, respectively [23]. However, the relationship between VEGF and poor prognosis was not verified in this study [23].

.Our study mainly discussed the heterologous sarcomatous components, the surgical approach, radiotherapy and chemotherapy, and chemotherapeutic drugs on the prognosis of OMMMT. The histological feature of OMMMT is that it contains both cancerous and sarcoma components. According to the differentiation of sarcoma components, OMMMT can be divided into homologous and heterologous subtypes. Some scholars believed that heterologous subtype was related to poor prognosis [57, 58], which might be due to the low response rate of chemotherapy in patients with sarcoma as the main component [59], while others considered that there was no difference between the prognosis of the two types [22]. By analyzing 89 patients in this group, the results supported that heterogeneity did not have an influence on the prognosis. In view of the fact that our sample size is larger than that of previous studies, the present evidence tended that the heterogenous components of sarcoma did not affect the prognosis of OMMMT.

Ovarian tumor cell debulking surgery and chemotherapy are often used to treat OMMMT, but there are no randomized clinical trials to support this method [12]. The OOTCDS included total hysterectomy, bilateral oophorectomy and salpingectomy, appendectomy, omentectomy, pelvic and abdominal paraaortic lymph node dissection and other tumor volume reduction [9]. Satisfactory cytoreductive surgery is defined as the maximum residual lesion diameter less than 1 cm after operation. Research confirmed that the OOTCDS could significantly delay the recurrence time of OMMMT [2]. Rutledge et al. analyzed 31 patients with advanced stage (III) carcinosarcoma who underwent surgery, and found that the size of residual tumor after surgery significantly affected the survival rate of patients [60]. In addition, many studies had suggested that OOTCDS could significantly prolong the median survival time of OMMMT patients [61, 62]. Our study confirmed that the median survival time of patients who took the OOTCDS was significantly prolonged (43 months VS.16 months).

Due to the lack of large-scale clinical studies, first-line chemotherapy regimens of OMMMT are inconclusive. Our study confirmed that postoperative chemotherapy was an independent protective factor for the prognosis of OMMMT (*P* < 0.05). Research indicated the 6-month relapse-free survival rates of 83, 36, 60% and 3-year survival rates of 67, 10 and 60% in the paclitaxel/carboplatin (TC) regimen group, other platinum-containing regimen group, and non-platinum regimen group, respectively, suggesting that the more satisfactory efficacy of TC regimen and platinum-containing regimen in OMMMT patients [15]. Brackmann et al. reported that TC treatment group had significantly longer progression-free survival compared to the isocyclophosphamide + paclitaxel treatment group (17.8 vs. 8.0 months), but overall survival was similar for all treatment groups [63]. Rutledge et al. retrospectively evaluated treatment effect of the platinum + isocyclophosphamide combination group versus the TC combination group, the evidences declared that the former one had longer progression-free survival and overall survival than the latter one [60]. Besides, the efficacy of other chemotherapeutic agents, such as sorafenib, topotecan, gemcitabine and docetaxel was not significant [64]. After comparing the effect of various chemotherapy regimens on the overall survival time, our study find that the optimal chemotherapeutic agent was platinum-based chemotherapy. In addition, the combination of platinum and taxanes is better than the combination of platinum and biological alkylating agents. Therefore, more clinical trials are needed to reconfirmed the optimal chemotherapy regimen to improve the medical efficacy. Radiotherapy is less frequently used in OMMMT. Blanco Suarez et al. reported a case of OMMMT with a recurrence of a large pelvic mass (14 cm) as the main manifestation after surgery, and the administration of chemotherapy combined with fractional radiotherapy resulted in a significant reduction in the size of the mass and effectively prevented the progression of the tumor [65]. However, there were no unambiguous evidences about whether the application of radiotherapy in patients with early OMMMT could improve the prognosis [64, 66, 67].

Due to the poor treatment effect of other chemotherapy drugs, biologically targeted therapies have become a new research direction in recent years. Programmed cell death ligand 1(PD-L1) is expressed in a variety of tumors and binds to its receptor programmed cell death protein 1(PD-1) to activate the PD-1/PD-L1 signaling pathway and inhibit the activity of effector T cells to produce immunosuppression. Blocking this pathway can enhance the endogenous anti-tumor immune response of the body [68]. Recent article proposed that CD8 + T cell and negative expression of PD-L1 in the mesenchymal component of OMMMT appear to be associated with a better prognosis, which seemed to confirm that the PD-L1 inhibitory pathway regulated tumor-infiltrating CD8 + T-cell responses in OMMMT [69]. Conversely, there is evidence that PD-L is a favorable factor for prognosis [70]. This may be attributed to the fact that response to PD-L1 blockade required a preexisting adaptive immunity in tumors, and tumors without tumor-infiltrating lymphocytes failed to respond [71]. Therefore, it is hypothesized that the PD-1/PD-L1 signaling pathway may be a new immunotherapy target for OMMMT. Research showed that trastuzumab-emtansine(T-DM1) inhibited the growth of uterine and ovarian carcinosarcomas cell lines with human epidermal HER2 overexpression in vivo and in vitro [72]. In addition, SYD985, a novel duocarmycin-based HER2-targeting antibody-drug conjugate, was more active than T-DM1 and had a stronger inhibitory effect on uterine and ovarian carcinosarcomas cell lines [73]. Research reported that solitomab was a bispecific single-chain antibody construct targeting epithelial cell adhesion molecule on uterine and ovarian carcinosarcomas cells and might be an effective drug [74]. Therefore, targeted therapy of OMMMT is worthy of further exploration.

In brief, OMMMT is a rare malignant tumor with rapid progress and poor prognosis. At present, the OOTCDS and adjuvant chemotherapy can improve the prognosis of patients. The optimal chemotherapy regimen is platinum-based scheme at present. In addition, the combination of platinum and taxanes is better than the combination of platinum and biological alkylating agents. Targeted therapy might be a new research direction in the future. Besides, protein omics, a rapidly evolving branch of science, may be essential for the discovery of novel therapeutic target and treatment decisions [75]. Protein omics techniques such as mass spectrometry and protein array analysis have been used to investigate the protein omics characterization of ovarian cancer, protein biomarkers, and response to drug therapy for ovarian cancer [76]. In a recent study, mass spectrum was applied for quantitatively comparing the protein of ovarian cancer tissues from patients who were sensitive to platinum and those who were resistant to platinum. It was found that cancer/testis antigen45(CT45) could regulate the activity of phosphorylase-4 to increase the sensitivity of platinum, and the CT45-derived human leukocyte antigen peptide segment could also enable tumor cells to be recognized and killed by T cells [77]. Zhang et al. proposed that the high expression of thioredoxin domain-containing protein 17 was related to paclitaxel resistance, which could increase the formation of autophagy in ovarian cancer cells and reduce the lethality of paclitaxel [78]. At present, there is no protein omics analysis of OMMMT. Thus, focusing on the specific protein omics characterization of OMMMT in the future may help to identify new therapeutic schemes.

## Supplementary Information

**Additional file 1.**

**Additional file 2.**

## Data Availability

All the data used to support the findings of this study are available from the corresponding author upon reasonable request.

## Abbreviations

OMMMT: Ovarian malignant mesoderm mixed tumor
FIGO: International Federation of Gynecology and Obstetric
CNKI: China Knowledge Resource Institute
OOTCDS: Optimal ovarian tumor cell debulking surgery
VEGF: Vascular endothelial growth factor
CT: Computed tomography
PET: Positron emission tomography
AFP: Alpha-fetoprotein
AMH: Anti-Müllerian hormone
HER2: Human epidermal growth factor receptor 2
TC: Paclitaxel/carboplatin
PD-L1: Programmed death ligand 1
T-DM1: Trastuzumab-emtansine
CT45: Cancer/testis antigen45
